# The Corn-stalk Disease in Cattle

**Published:** 1889-07

**Authors:** Frank S. Billings

**Affiliations:** Director of the Patho-Biological Laboratory of the University of Nebraska


### *THE CORN-STALK DISEASE IN CATTLE.*

By FRANK S. BILLINGS,

Director of the Patho-Biological Laboratory of the University of Nebraska.

The name "Corn-stalk Disease," though not exactly expressing what is desired, has been selected for this etiologically new and entirely unknown disease in cattle, because practical experience has shown that the eruption is generally connected with the turning of cattle into the stalk-fields in the later Fall and Winter months,—a sort of lazy man's method of gleaning up the remnants. As will be conclusively shown, and conforming to the actual facts, it is not the corn-stalks, but the leaves and tender topshoots, which indirectly cause the peculiar disease in cattle (and other herbivorous animals?) of which we are about to treat. Anyone who carefully observes cattle when turned into stalk fields, will see that they seldom meddle with the hard, dry, bulky stalk, but that, as they pass along, they pull off the leaves and the tender top-portion of the stalks. The same complaint has also been attributed to the smut in corn, but a little reflection, combined with observation, would show that this idea is entirely groundless, as has been demonstrated by experimentation. Another equally absurd idea, to which we shall soon allude in detail, is that lack of water is the cause of this malady. How long this disease has afflicted cattle in the United States, it is impossible to determine, nor can we make any estimate as to the amount of loss it annually causes the farmers of the great corn-raising States of the West, though it is by no means limited to them; still, it can be safely said that this malady causes more loss in cattle to the Western farmer than all other causes combined, not excepting abortion. The more one becomes engaged in the scientific investigation of these questions, the more does he become convinced of the pressing necessity of the collection of exact statistics upon the losses in our live-stock annually, and disgusted with the farce played in nearly all our States by the totally incompetent lay cattle or live-stock commissions, and the utter inadequacy of the present system of education in the veterinary schools of the country to prepare men for these most responsible and, if adequately filled, highly honorable positions. It should never be forgotten that it is the qualifications of the individual that make a position honorable, and not the position the individual. Persons totally ignorant of the advanced position of modern hygienic medicine, and the methods of exact scientific investigation, must, of necessity, be utterly incompetent to comprehend the true responsibilities or nature of the work connected with the preservation of our live-stock from preventible diseases. As will be well illustrated by the disease in question, such persons are totally dependent on the musty and antiquated ideas of earlier writers for their knowledge, and absorb them unreflectingly as " Gospel truths," according to the historical reputation of the author, and neither have the knowledge, ability, nor desire to think for themselves. According to such people, the "*has been*" must always be THE *true*. They never think to weigh it by the living present. Facts presented to their eyes are utterly ignored, while the truth is entirely hidden by a mighty respect for the ignorance of the past when covered with the glamour—sometimes deserved—of authority.

So much for an introduction.

The first allusion to this disease, which is to be found in the literature at my command, is an article by Prof. John Gamgee on the "Ill Effects of Smut in the Feed of Farm Animals," (Report of the Commissioner of Agriculture on the Diseases of Cattle in the United States, Washington, 1871, p. 73,) who says:

"The opportunity presented itself last Fall for an inquiry as to the manner in which the smuts which attack plants may affect animals. The latter part of 1868 was, throughout America, very wet; a large amount of corn became smutty: that is to say, was attacked to a very serious extent by *ustilago maidis*, and reports reached me from the West and South that cattle were dying in large numbers from a mysterious malady, the origin of which was unknown. From Mills county, Iowa, I was informed, late in November, that about the twelfth of the month there was a fall of snow six inches deep, and that the cattle, which usually roam at large on the prairies, were taken in by all the better farmers who had their corn gathered, and *turned into the stalk-fields. In about eight days the cattle began to die, all presenting the same symptoms.* My informant lost four out of nineteen head in fourteen days.

"Personal inquiries among gentlemen from different parts of the United States enabled me to trace the malady in Western Virginia, Illinois, and the Carolinas. It is much to be regretted that accurate information as to the extent of the losses, and localities affected, cannot be secured.

"There are other circumstances under which cattle die from eating corn. The stalks, very late in the season, are apt to become very hard and indigestible, and, without a free admixture of grass, which the early frosts kill, or other food, they are apt to cause indigestion and death. This is an observation that has often been made in America. The facts published with regard to the prevalence of a malady among cattle in America, caused by eating smutty corn, are very few. If, however, the real cause of the so-called dry murrain had been correctly recorded, there would be no difficulty in demonstrating that the condition of the corn-fields has had much to do with developing this disorder.

"The Department of Agriculture has received information of the death of cattle from eating smutty corn in Hampshire county, Massachusetts; also from Whitley county, Indiana, where seven head of cattle out of fifty died, probably from smut in the cornfield in which the herd ranged.

"From Storey county, Iowa, it is reported that, last November, a disease appeared among herds recently turned into cornstalk fields. The disease is evidently the dry murrain. *Post mortem* examination showed the mucous membrane of the stomach to be highly inflamed. It is evident that the disease is generated in the stalk-fields, and probable that it is produced by gorging the stomach when first turned into the stalks, after being confined to the wild, frost-bitten prairie-grass, and lack of sufficient water. A few cattle died of dry murrain in Audubon county, in the same State, supposed to be caused by smut in the corn-stalks. A few head were lost from the same cause in Calhoun county, and many are reported to have died in Marshall county. We are, however, informed from Sac county that many cattle died in December,—cause unknown,—some suppose from eating smutty corn, but that has been disproved. It is to be regretted that more is not stated with regard to the reasons which led persons to doubt the effects of the smutty corn. Even in New York, little credence was given to the action of smutty corn at first, but careful inquiry proved that, after all, it was the cause of the dry murrain in the Fall of 1868. From Dakota county, Nebraska, we learn of dry murrain from the same cause, whereas from Shawnee county it is reported, and no doubt correctly, that the same disease has been noticed among cattle fed on prairie-hay cut after frost."

Let us now subject these views of Mr. John Gamgee to critical analysis. It is self-evident that the above statements are all of an *a priori*—that is, without proof,—character. Mr. Gamgee had actually neither practical knowledge of, nor experience with, that of which he was writing. Yet, it seems highly probable that he is the responsible authority for the prevalence of these ideas in the minds of the American veterinarians of to-day, and in our literature. In fact, Mr. Gamgee accepted, and but repeated, the idea prevailing more or less among cattle men of the time, who, on observing disease sometimes occurring in their cattle when turned into the stalk-fields in the Fall and Winter, and seeing, or perhaps supposing, smutty corn to be present, and nothing else to attribute the trouble to, jumped to the conclusion that the smut must of necessity be the cause. They, nor Mr. Gamgee, did not stop to think that hundreds of other cattle in adjoining fields, or those near, were also turned into stalks, and at the same time of the year, and still did not become ill; nor did they or he go into those fields and examine for the existence of smut. A little common sense and critical consideration, combined with exact observation, should demonstrate to any practical man that dry corn-stalks of themselves could never have caused the trouble, nor could the smut; for it is a well-known fact that many cattle acquire a decided taste for corn-smut, and even seek it out, and no evil results have ever occurred therefrom.

Now a word as to the term "dry murrain."

It is one of those meaningless expressions which have crept into veterinary literature from the days of crude empirical ignorance, and been fostered by men whose education should have taught them better than to use it. The word "murrain" is an old English expression used to denote the existence of any malignant disease among a considerable number of cattle, and was once especially used in connection with the rinderpest and the contagious lung-plague. It had no specific meaning. The word "dry" has been prefixed to it simply to designate the fact that the contents of the third stomach was in a more or less abnormally "dry" condition. We shall refer to this question again later on.

Let us now turn to Mr. Gamgee's testimony on the smut question:

SMUT NOT THE CAUSE OF THE DISEASE.

Mr. Gamgee says that he was—

"Anxious to try some experiments on the action of pure smut on cattle. I employed a negro, in January, 1869, to go into the country and collect for me a large quantity of pure smut. It was rather late, and the rains had washed the most of it off the still standing stalks, but I obtained *forty-two pounds of excellent smut, free from adventitious matter.* On the 26th of February, purchased two cows in good health, aged about seven years. One cow was fed thrice daily one and one-half pounds of corn-meal and three ounces of smut, with as much cut hay as she would eat. The second had the same allowance, but wet. On the 7th of March, the amount of smut given in each feed was increased to six ounces. The cow fed on dry food lost flesh. On the 15th of March, the dose of smut was increased to twelve ounces three times a day. The cow on the wet food increased in condition; the other one lost. *In three weeks, the two cows consumed the forty-two pounds of smut.* They had a voracious appetite the whole time, and the only indication of a peculiar diet was a very black color of the excrement."

"Forty-two pounds of smut consumed by two cows in three weeks," or twenty-one pounds to each cow, with no evil effects whatever!

Does any sane person for a moment think that any cow, or steer, turned into a stalk-field, would possibly consume twenty-one pounds of smut in three weeks' time? The smut would have to be most fearfully plentiful, and the fields very large, which would yield twenty-one pounds of smut in the late Fall or early Winter months, and the cow most excessively busy. In fact, so busy would she have to be collecting that twenty-one pounds of smut in three weeks, that she would have no time to collect any other food, and probably die of starvation before she completed the job. If twenty-one pounds of smut had no ill effects upon either of two cows when forcibly fed upon it, we may be very sure that smut never killed a bovine when turned into a stalk-field, and hence Mr. John Gamgee's hypothesis is absolutely worthless. As to the frost-bitten grasses having killed cattle, were that a cause, more than half the cattle in our Western States would die every year. Such statements, or reasoning, from persons claiming education and practical experience, are absolutely sickening. Yet, they find credence in the minds of many veterinarians who, having neither powers of observation and reflection, and being absolutely devoid of common sense, rehash such mythical statements in the ears of anxious cattle-men, who are obliged to turn to them for advice.

It has been said that these misleading teachings of Mr. Gamgee have made their impression upon veterinarians and veterinary literature; in proof of which I quote from the "Second Biennial Report of the Board of Live-Stock Agents" of Nebraska, p. 23, where may be read the following:

"CATTLE IN CORN-STALKS.

"During the months of October, November, and December in each year, many reports are received by telegraph and mail that cattle are dying of some unknown disease. The State Veterinarian and assistants have investigated a great number of these reports, and find, in every case, that the cause of trouble was the feeding of corn-stalks left standing in the field. More cattle have died of this in this State than of all other causes combined. This disease is so easily prevented by good management and proper feeding, that the loss from this cause should be very small.

"Corn-stalk fields are a dangerous range for cattle, more especially so when the season has been favorable for ripening the stalks completely, and which has changed the starch and nutritious matters of the stem and leaf into an indigestible fiber, wholly valueless as food, and with a herd of cattle turned into such a field from off a dry range in the late Autumn, there can be but one result: over-gorging of matter which cannot be digested, impaction, and the loss of many valuable animals.

"The symptoms vary, but in nearly all outbreaks of this trouble, some of the following symptoms are noticeable: In some cases the animals are wild, with head erect, and eyes protruding, and a disposition to go where they please, or to attack anyone who may come in their way. Others stupid, dull, with low hanging head, more or less salivation, wabbling of the hind parts, knuckling of the fetlock-joints behind, inability to get up when down, stumbling, great nervousness, twitching of the muscles, loss of sensation, loss of appetite, rapid breathing, quick pulse,—many die. All of these symptoms are not noticeable in the same animal, but more or less of them are. As a preventive, cattle should not be turned into a field of dry stalks until after they have satisfied their hunger, and then only for a short time, particularly if the weather has been dry for some time previous. Allow plenty of salt and an unlimited quantity of water."

The above remarks were really directed against certain conclusions of my own, which unfortunately were, and are, based either upon exact experimentation or very close observation. Accompanied by similar clinical symptoms, we have in Nebraska, anthrax, which may occur at any season of the year, a disease which is generally spoken of as "Hydrophobia in Cattle," and which, according to popular rumor, and often most positive assertions, is always connected with the presence of a

" mad dog;" and the disease called the "mad itch," which is still different from either of the others. My having differentiated two of these diseases from anthrax, and Dr. Paquin, of Missouri, the " mad itch " caused these ignoramuses, technically speaking, to fall back on historical authority, equally incompetent with themselves, to decide the point at issue, and attribute them all to one and the same cause, that is, corn-stalks.

In the month of February, 1889, (not October, November, or December,) a very extensive outbreak of the " Corn-stalk Disease " occurred in the vicinity of Fremont, Nebraska, the following accounts of which appeared in the daily papers:

FREMONT, Neb., Feb. 7th.—John Delaney, a farmer living in Elkhorn township, five or six miles east of Fremont, is suffering the loss of a large number of cattle from his herd, from a disease which puzzles the veterinarians. He has lost fifty head to date. Yesterday he determined to investigate the cause of the fatality. He summoned State Veterinarian Osborn and Dr. Dulin, who made an investigation. They dissected several head of dead animals, and found the symptoms exactly the same in all of them. The fourth stomach was packed full of dry, hard food, and the surrounding organs and tissues were badly inflamed and feverish. The doctors were unable to diagnose the case, but they gave it as their opinion that it was not a contagious disease. The animals, when first affected, will bellow in a low, hoarse manner, shaking their heads. Within twenty-four hours after this they invariably (? B) die."

The *Tribune*, of Fremont, however, the eighth instant, gives us the results of this investigation, as follows:

" Dr. Osborn was seen this morning; speaking of the fatality among John Delaney's cattle, an account of which was given in the *Tribune*, he said that Mr. Delaney, and many of his neighbors, felt sure that the disease was a contagious one, but there was no reason or theory for any such supposition.

"'There is no contagious disease known to veterinarians which affects the third stomach of the cow,' said the doctor, 'and that was the seat of the trouble in every case I examined.' The third stomach, or manifold, was packed with dry food, which, taken in the fingers, crumbled like flour, and was so light it would blow away like chaff in the wind. The whole cause of the trouble is the condition of the food the cattle eat. The hay and corn-stalks are exceedingly dry, owing to the very dry Fall and Winter, and the cattle not having sufficient quantities of salt and water, congests as found. There has been some loss on the Hershey ranch, and a *post mortem* examination of the animals disclosed the same conditions. Mr. Hershey had lost sixteen head at the time I was there a few days ago."

Mr. Delaney writes me that his actual loss was—

| | |
|---|---|
| 10 steers, valued at | $300.00 |
| 12 calves " " | 120.00 |
| 39 cows " " | 974.00 |
| Total loss | $1,394.00 |

It will be at once seen that Dr. Osborn followed the authorities in his conclusions, but I will show that had he been competent to make a necroscopical examination and properly appreciate its teachings, that there was something else the trouble with the cattle than that "the third stomach was the seat of the trouble in every case examined." I will show that this condition of the third stomach was but the result and expression of another and most specific malady; that it was not diseased, but that its contents alone were the language of something else of an entirely different character.

This brings us again to that ridiculous and nihilistic term "dry murrain." It will be seen by reading Mr. Gamgee's remarks, and those of the exquisitely intelligent State Veterinarian of Nebraska, that they neither consider the corn-stalks to be of themselves the chief cause of the malady, but that insufficiency of water plays a still more essential role in its etiology, to which these later authorities have added want of "salt;" also, more "pure theory," as the people call it, greater unfounded, *a priori* reasoning, or conclusions, than the above, could not possibly emanate from the human brain. A moment's reflection, the most superficial acquaintance with the anatomy and physiology of cattle, should teach anyone that such a "dry" condition of the third stomach is absolutely impossible under the conditions mentioned. Anyone with a grain of common sense should know that the gastric arrangement of bovines is similar to that of the camel, the first stomach being the receptacle for vast amounts of both food and water, thus supplying a surplus of such materials over the almost hourly requirements of other animals in comparison; which fact has been taken advantage of by man in countries where extensive desert tracts prevail, barren of either food or water, the camel being able to go several days without either, but more especially water, without any intolerable inconvenience. It has often been recorded by travelers that when the water supply of the caravan had given out, that a camel had been killed in order to get a supply of that vitally necessary material to the preservation of human life. In this regard, camels and cattle are exactly alike, and under such conditions as prevail in our agricultural districts, it is beyond the range of possibility that cattle should go so long without water as to produce any effect whatever upon the dryness of the contents of the third stomach or manifold. The dryness in the third stomach mentioned by Dr. Osborn was not due to an insufficient supply of water, far less salt, but to an extremely high and prolonged rise of temperature in the animals in connection with an acute blood-infectious, septicemic disease, which condition would have been at once revealed by the use of a thermometer upon some of the diseased living animals, and which any competent person would have immediately recognized in the character of the necroscopical lesions.

### LACK OF WATER OR SALT CANNOT POSSIBLY CAUSE THE CORN-STALK DISEASE.

Having previously shown the impossibility of these two factors playing any role in the causation of this disease from theoretic and anatomical and physiological grounds, I wish now to present practical evidence of the correctness of my assertions, from the observations of a farmer, in the form of one of the most remarkably intelligent and observant letters it has ever been my fortune to receive from a live-stock breeder.

Mr. Samuel McKelvie, of Fairfield, Nebraska, wrote me under date of April 1, 1889:

"During the past Fall and Winter I have lost nine head of cattle from the stalk-field trouble, whatever it is. I have lost all of these from one field of forty acres, and I have noticed closely and have thought of different causes and theories, but the last one that died knocked all out of me. I am at a loss to know what the cause is, unless some disease is in the field. I will give you the particulars as near as I can, and if it is not asking too much of you, I shall be very glad to hear your ideas about it.

"In November last, I commenced feeding my cattle (about forty head) shock corn from this field of forty acres, of which I have spoken. They then ran in a pasture of prairie of about 200 acres. One day I noticed them gathered in a bunch in the pasture, and on investigation found one of the number dead and swollen tight. It was a calf that had been weaned some three weeks, and in excellent condition. I did not examine it. In about two weeks, or about the last of November, I got the corn all gathered out of said forty acres, of which about three acres had been cut up in shocks and the rest left, and the corn shucked off. On Sunday, I turned my cattle in about one and a halt hours in forenoon. The balance of the day they were in a lot with straw (oat straw) in a rack, with plenty of water and salt in their yard, so they could get it if they wished. On Monday, I turned them back in said field, in forenoon, about two hours. On Tuesday, turned them in about two hours in forenoon, and two hours in afternoon. Wednesday morning I left home early with orders to turn the cattle in stalks same as day before. My little boy went out after breakfast and found two dead and one sick. The hired man proceeded to doctor the sick one, and while working at it noticed another sick. The first one soon died; the second lived until next day. Stood up all the time; must have dropped dead off his feet. We saw him a few minutes before death, standing up. They did not bloat until just at the last, and some did not bloat any. This last steer grunted and seemed to be in great misery. Of these four that died, three were yearlings, and the other a small two-year-old.

"Now, as there was plenty of water in two tanks in the yard, I thought that the larger cattle had kept these away until the dry feed had packed in the stomach and killed them. So I turned those that were left back the next day two hours in the forenoon, and brought them back into the yard, and stayed with them until all drank. Turned them back in the evening for same time, and saw that all drank when they came out. Next day I turned them back, or looked through them before breakfast, and they were all right. Sent the boy after breakfast to turn them into the stalk-field. While in at breakfast one of the best two-year-old steers had taken sick and left the rest that were about the straw-rack and went below the barn and lay down. In about two hours from the time the boy went to turn them into the field, I went to bring them in and water them. On going to the barn to get a horse, I noticed the one spoken of lying below the barn almost dead. He would stretch out and tremble and grunt, and could only raise his head off the ground to his side. I sent to the field at once for the cattle, and stayed with the sick steer. When the cattle came in I counted them and found one short. I sent back to the field, and the report came back that another good two-year-old was almost dead in the stalk-field. I went up and found him in about the same shape as the one just described, and they both died in about two hours. When I came back from the field, which was one-third of a mile from the yard, the cattle that had been put into the yard were most all lying down. I went among them and noticed one that did not seem just right. I went to him and scared him up, and it was all that he could possibly do to get up. He was just taken. When he got up he went staggering off, stepping high; his limbs seemed to be numb, and his sight affected. I drove him around, and gave him a small dose of saltpetre. The more I drove him, the better he seemed to be. He walked all right, but seemed to be in great misery, and stood up and grunted until dusk, when he lay down, and died about 10.00 P. M. This, too, was a large two-year-old steer. *Now, here were three of the very best of my cattle and my water theory all gone up.* I took them out of this field and turned them into another. They cleaned it up, not any dying; then turned them into another, which they cleaned up without any trouble; turned them back into the forty-acre field in February to clean it up, thinking the Winter had by this time got away with the trouble. They ran there two or three days, and one died. I then took them out of that field, and kept them out, and made up my mind that I had just as well feed strychnine as leave them there."

Can more strong and positive practical testimony be given of the utter fallacy of the "dry murrain," short-of-water-and-salt hypothesis?

Upon the same subject, a Mr. W. E. Thorne, of Bladen, Webster county, Nebraska, writes under date of March 25, 1889:

"I have lost five head of cattle from running in corn-stalks this Winter; two on the 26th of December, the others at various times since; the last one affected was about two weeks ago, but it recovered."

As to the salt-and-water business, he says:

"*I took especial care to salt and water my cattle, and I know that there was no lack of either.*

"After death, I opened and examined the animals, but could not satisfy myself as to the cause of their death. *The first that died had no impaction of the third stomach whatever*, but in one or two of the later ones the contents were pretty dry and hard. When first taken, they were very sick, and I thought there must be some substance in the stalks of a poisonous nature. The di-ease has been quite prevalent in various places about here, some fifty to seventy-five head having died within a radius of six or eight miles of this place."

ARE HORSES ALSO SUSCEPTIBLE TO INFECTION?

Upon this subject I am in receipt of the following:

"BYRON, Neb., March 18, 1889.

"*My Dear Sir*—About the 10th of December, 1888, my brothers and myself fenced off about 200 acres of stalks, and turned into them a lot of colts, leaving them there but a short time each day. The stock did well until about the first of January, when we lost seven colts within the same number of days. They appeared to become crazy and blind, most of them falling dead while running. We lost a great many colts in this way in Thayer county, Nebraska, and Republic county, Kansas.

"Yours, JOHN A. FISHER."

Was it the disease of which we are treating?

That there is a very direct connection between ensilage and a disease in horses, has been known for a long time, though it is rare. The following quotation is interesting in this connection:

"WAS IT SILAGE OR SMUT THAT KILLED THE HORSES?

"*To the Farmers' Review, Chicago, Ill., March*, 1889*:* The subject of feeding silage to horses, that you have made so interesting in late numbers of the *Review*, is one of great interest, and more so as relates to the death of some horses, said to have died from the effects of the silage. The case from Kentucky, I think, will bear a little closer investigation. A private letter received by myself from a party in Kentucky who had lost twelve horses from eating silage, said the surgeon had pronounced it cerebro-spinal meningitis, from silage-eating. A noted veterinarian tells me that this disease is so rare that it would be impossible to have twelve cases closely associated, and gave it as his opinion that the silage was made from corn heavily charged with smut (ergot), and the conditions of the silage-making were such that the formation of smut-spores went on, permeating the entire mass, and by feeding the horses 'all they would eat,' they were killed by paralysis of the spinal cord. That might easily be mistaken for meningitis. What makes me think this is so, is from a statement in this letter, to the effect that the silage was badly spoiled along the sides, and occasionally layers would be found badly damaged. This would show that there was an entry of air from some source; that would cause the decay, and the smut-spores, taking advantage of this, would propagate where the weather is no colder than it has been near Louisville, Ky., this Winter. Hereabouts horsemen feed horses, broodmares, and colts large amounts of silage; not 'all they will eat,' but reasonable rations, and not a case have I heard of unfavorable. But, in silo-filling, stalks that are smut-laden are pitched overboard, not put in the silo, and smut-balls are twisted off from less producing ones. The idea is to have good silage go into the pits. Now, possibly, this smut business may not have any existence in fact, so far as it relates to this Kentucky silo, and this guess about it may be all wrong. By the way, this gentleman who writes me from Crescent Hill, Ky., about this matter, instances a neighbor of his, twelve miles distant, who has a silo, and has lost a similar number of horses from the effect of silage, and remarks that 'silage is killed in that section.' I am pleased to see Prof. Henry's remarks in connection with this matter, for what he says is authority and has great weight. His idea that a little acid in the silage makes it more favorable for digestion, recalls some experiments East this Winter, when a certain amount of cider-vinegar was mixed with the dry feed, and with most beneficial and paying results. At the late Silage Congress in Cleveland, Dr. Ashmun, the Health Officer of Cleveland, testified that there was no danger or damage in a reasonable amount of acid in silage, and Dr. Stewart affirmed that, without a certain amount of lactic and acetic acid, there could be no digestion.

"JOHN GOULD."

I have personally seen quite a number of cases exactly corresponding to the above, when horses were fed on corn from a silo, some years ago, in Massachusetts, but had no conveniences at the time for properly investigating it. Other cases have also been reported in different parts of the country, but no mention is made whether cattle were fed the same material or not, or whether any died therefrom. A gentleman of much experience also tells me that corn-fodder will affect sheep and goats, but not hogs. That hogs will not become infected, is well known. At Mr. Delaney's they were with the cattle all the time, and also in another outbreak, to which we shall soon refer; and I have endeavored to kill hogs by the subcutaneous injection of such extreme doses as 6 fl. c.c. ms. of a very malignant, pure culture, but in no case have the hogs shown any signs of illness.

### PERSONAL EXPERIENCE WITH THE CORN-STALK DISEASE.

I much regret to have to say that I have never been able to see a single case of this disease as it occurs under natural conditions, my engagements in the laboratory—having no assistance—being of such a nature, and the outbreaks occurring at such a distance from Lincoln, that it has been impossible for me to make either clinical or microscopical examinations; hence my notes on this very important subject must of necessity be most meager and unsatisfactory, but to no one so much so as to myself. The first case with which I had any connection was of a most unique and unexplainable character, and remained a complete mystery to me until my investigations were completed on the Delaney outbreak, at Fremont, this year. It still remains mysterious in many ways, but it was from this case that I first obtained the etiological organism, and it is only from comparison of my notes of its methods of development in and on different media that I am enabled to recognize it as the same disease which occurred at Fremont, a little over a year subsequently.

### A SINGULAR CASE OF CORN-STALK DISEASE AT AMES, NEBRASKA.

On January 6, 1888, Dr. W. A. Thomas, a veterinary surgeon, then in the employ of the State Live-Stock Commission as an inspector, came into the laboratory with two bottles—one containing the blood and the other some pieces of the organs of a steer which had died very suddenly at the feeding station of the Standard Cattle Company, at Ames, where he had been sent to inspect a number of horses on account of glanders. As to the steer all he could say was that it was considered well the night before, at least its condition did not attract any attention, and that it was found dead on the men's going to the stable the next morning.

Knowing my interest in all such cases, Dr. Thomas hastily opened the animal and brought me the material mentioned, only reporting "the blood to have been fluid and of a dark color, the liver swollen, anemic, and of a dirty yellowish-gray-brown color; the spleen much swollen and weighing five pounds, and the kidneys swollen, cortical substance gray-red and anemic, while the medullary was bright red."

I at once made a microscopic examination of the blood by covering glass specimens, and cannot express the astonishment and perplexity which came upon me on seeing an apparently pure condition of innumerable microörganisms having the polar staining and belted appearance, as well as the size and form of those I had already discovered in the Southern cattle plague (Texas fever), and which I had demonstrated beyond the possibility of question to be the specific cause of that disease. These phenomena of resemblance were still more strengthened by the fact that like the etiological organism of the Southern cattle plague, this germ colored much more sharply in a fuchsin solution than in either a blue or violet, which fact is an essential point of differential value between these two organisms and that of the true swine plague, which colors better in the blue or violet tinctions than in fuchsin.

That the disease could possibly be the Southern cattle plague, seemed to be shown by the fact that all and every experience in the latter disease contradicted its appearance in our Northern climate in the midst of the Winter. Again, I soon found that pure cultures of the new germs were rapidly fatal to rabbits, while I have thus far found these rodents immune to the action of the etiological organism of the Southern cattle plague in any reasonable doses injected under the skin. I also found essential points of differentiation in the development of the two organisms on solid media.

The wherefore of the death of this steer, in so singular a manner, and among over a thousand others, all of which were, presumably, fed in the same manner and with the same materials, was then a great mystery, and although we now know how it must have occurred, we still cannot understand why the outbreak was limited to a single animal, unless it happened to eat all the infected stalks and leaves on the place; possibly a single stalk and the leaves attached to it.

(*To be continued in the August number.*)

